# Supplementary material for: A new stem sarcopterygian illuminates patterns of character evolution in early bony fishes
Source: Nat Commun. 2017 Dec 5;8:1932. doi: 10.1038/s41467-017-01801-z (PMC5715141; doi:10.1038/s41467-017-01801-z)
Supplement: Supplementary file 1 — Supplementary Information [file 41467_2017_1801_MOESM1_ESM.pdf]

Supplementary Figures

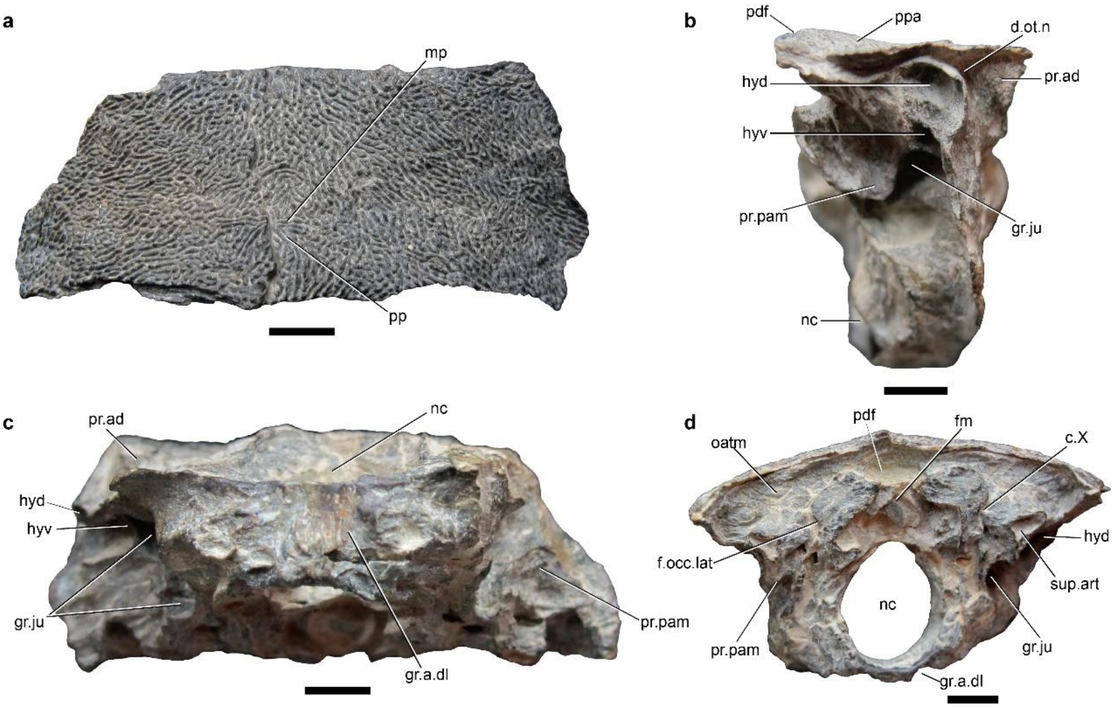

Supplementary Figure 1 | The skull of *Ptyctolepis brachynotus* gen. et sp. nov. (IVPP V23386). Specimen in dorsal (**a**), right lateral (**b**), ventral (**c**) and posterior (**e**) views. c.X, vagus nerve; d.ot.n, dorsal branch of the otic lateral line nerve; fm, foramen magnum; f.occ.lat, lateral occipital fissure; gr.a.dl, groove for lateral dorsal aorta; gr.ju, groove for jugular canal; hyd, dorsal hyoid articular area; hyv, ventral hyoid articular area; mp, middle pit-line; nc, notochordal canal; oatm, attachment area for trunk musculature; pdf, posterior dorsal fontanelle; pp, posterior pit-line; pr.ad, antero-dorsal process; pr.pam, parampullar process; sup.art, articular area for suprapharyngobranchial. Scale bar, 5mm.

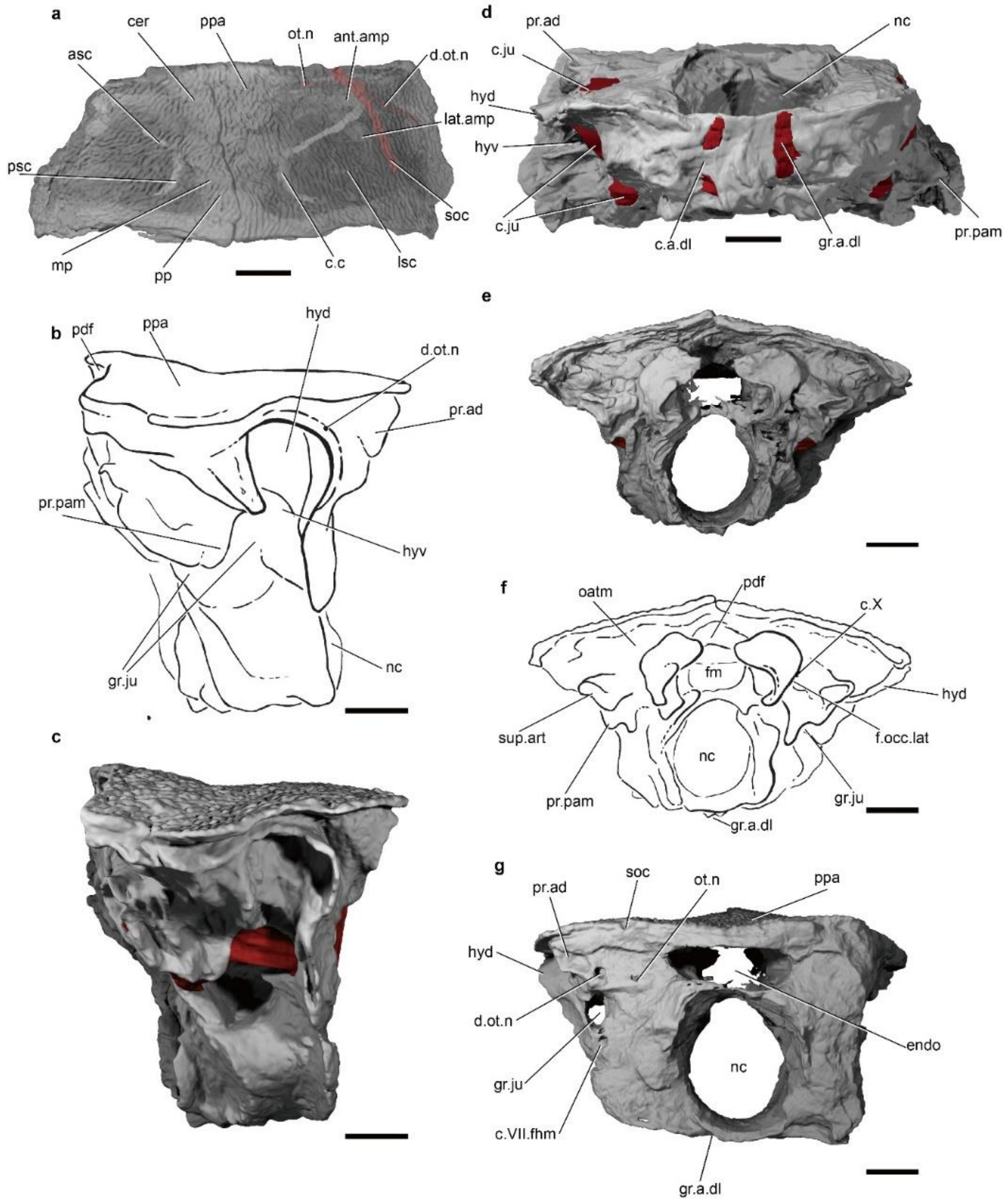

Supplementary Figure 2 | The skull of *Ptyctolepis brachynotus* gen. et sp. nov. (IVPP V23386), based on high-resolution CT. (**a**) Transparent skull roof in dorsal view, showing the neurocranial endocast (in grey) and vasculature (in red). Rendering (**b**) and interpretive drawing (**c**) of skull in right lateral view. Rendering (**d**) and interpretive drawing (**e**) of skull in ventral view. Rendering of skull in anterior (**g**) view. ant.amp, ampulla of anterior semicircular canal; asc, anterior semicircular canal; c.a.dl, canal for lateral dorsal aorta; c.ju, jugular canal; cer, cerebellum; c.c, crus commune; c.VII.fhm, hyomandibular branch of facial nerve; endo, endocavity; lat.amp, ampulla of lateral semicircular canal; lsc, lateral semicircular canal; ot.n, otic lateral line nerve; ppa, postparietal; pr.pam, parampullar process; psc, posterior semicircular canal; soc, supraorbital canal. For other abbreviations see Supplementary Figure 1. Scale bar, 5mm.

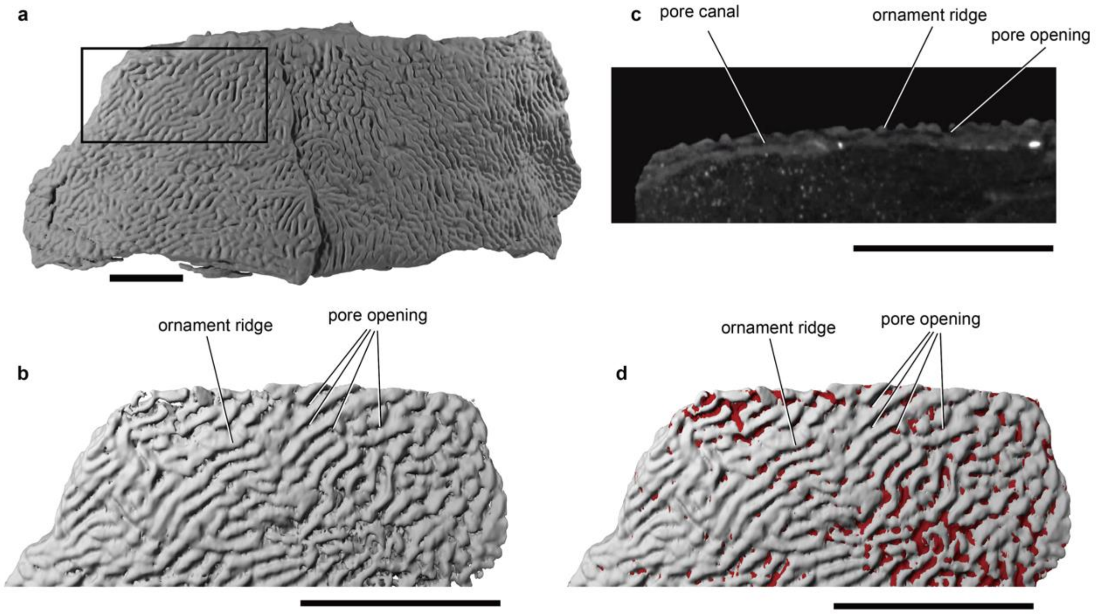

Supplementary Figure 3 | Dermal ornament and histological structure of *Ptyctolepis brachynotus* gen. et sp. nov. Red, pore canal network. (**a**) Overview of skull roof showing area of interest. High resolution rendering of dermal ornament in dorsal view without (**b**) and with (**d**) pore canal network infilled. (**c**) Tomograph through skull roof showing pore canals opening on the side of ornament ridges via pore openings. Scale bar, 5mm.

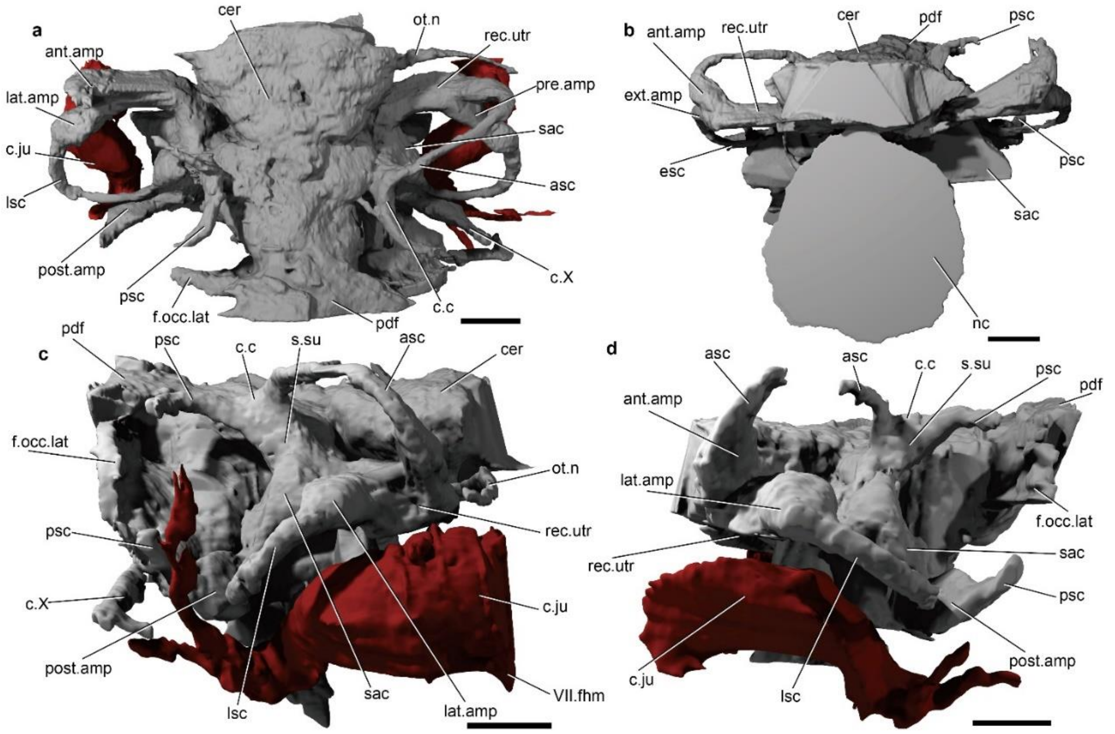

Supplementary Figure 4 | Digital neurocranial endocast restorations of *Ptyctolepis brachynotus* gen. et sp. nov. Dorsal (**a**); anterior (**b**); right lateral (**c**) and left lateral (**d**) views. Red, jugular canal. ant.amp, ampulla of anterior semicircular canal; asc, anterior semicircular canal; cer, cerebellum; c.c, crus commune; c.ju, jugular canal; f.occ.lat, lateral occipital fissure; ot.n, otic lateral line nerve; pdf, posterior dorsal fontanelle; lat.amp, ampulla of lateral semicircular canal; lsc, lateral semicircular canal; post.amp, ampulla of posterior semicircular canal; pre.amp, preampullary canal; psc, posterior semicircular canal; rec.utr, utricular recess; sac, sacculus; s.su, sinus superior; c.VII, facial nerve; c.X, vagus nerve. Scale bar, 5mm.

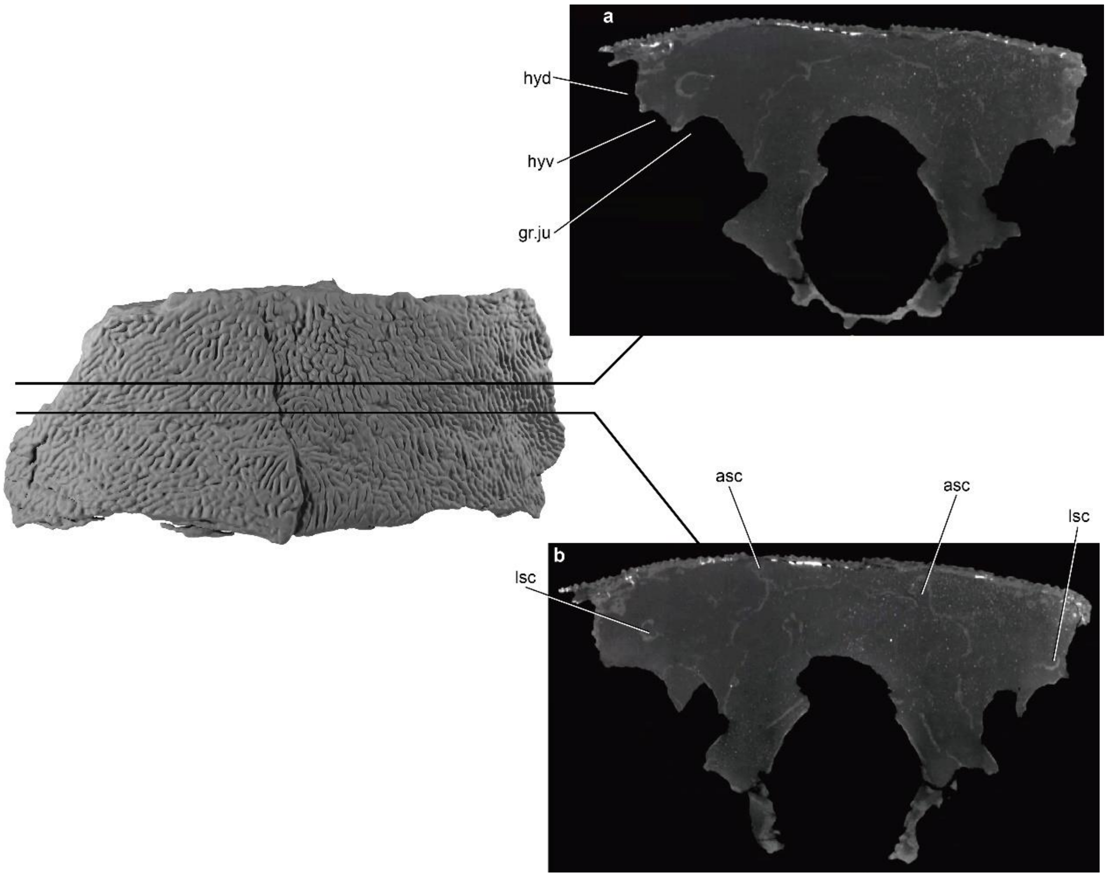

Supplementary Figure 5 | Tomographs through the skull of *Ptyctolepis brachynotus* gen. et sp. nov. Red, pore canal network. (**a**) Section through articular areas for hyomandibular positioned dorsal to jugular groove. (**b**) Section through the labyrinth showing narrow diameter of semicircular canals. asc, anterior semicircular canal; gr.ju, groove for jugular canal; hyd, dorsal hyoid articular area; hyv, ventral hyoid articular area; lsc, lateral semicircular canal. Not to scale.

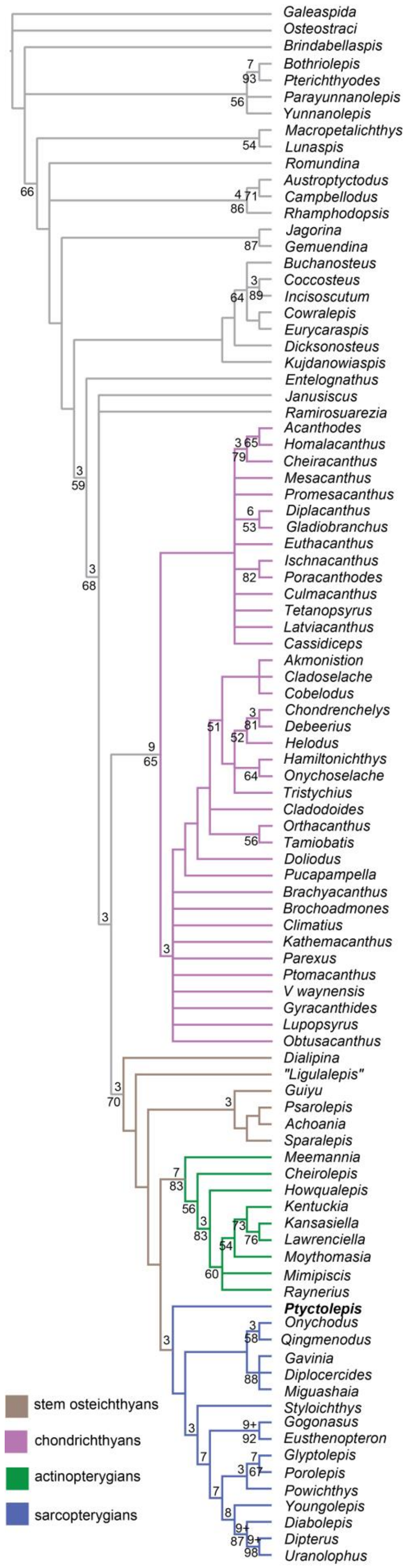

Supplementary Figure 6 | Results of maximum parsimony analyses. Strict consensus of the 861680 shortest trees (802 steps) for 94 taxa and 278 equally weighted characters. Digits above nodes indicate Bremer decay indices above 1. Digits below nodes indicate percentage bootstrap support above 50%.

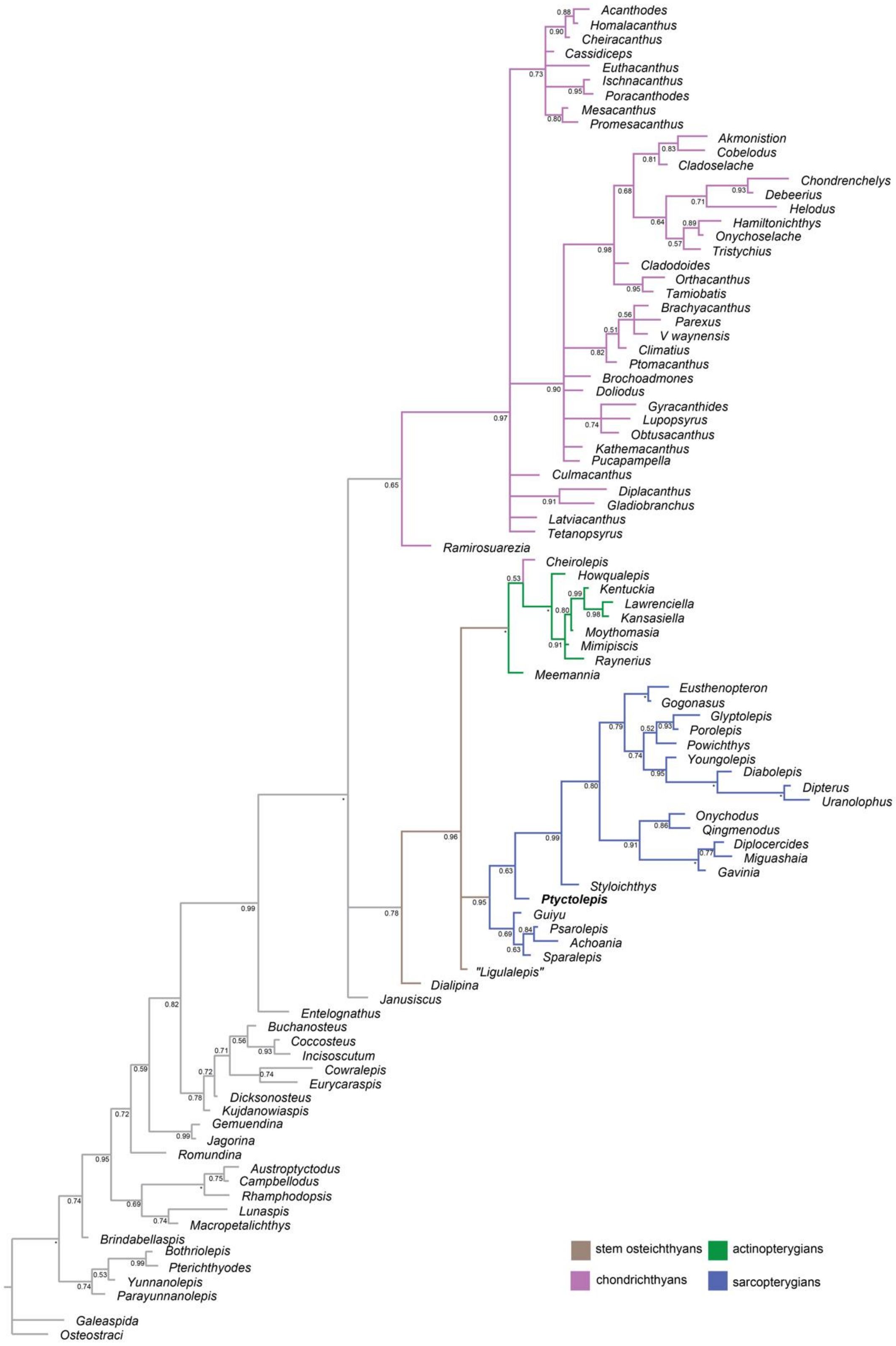

Supplementary Figure 7 | Results of Bayesian inference analyses. Numbers at nodes represent posterior probability support; asterisks represent a posterior probability of 1.

Supplementary Table 1. Taxa included in the analyses with associated references

Taxon	References
Galeaspida[1-3]	Donoghue et al. 2000; Wang et al. 2005; Gai et al. 2011.
Osteostraci[4-6]	Denison 1947; Janvier 1985; Sansom 2009.
Acanthodes[7-12]	Gross 1947; Miles 1968, 1973a; Coates 1994; Valiukevicius 1995; Davis et al. 2012.
Achoania[13,14]	Zhu et al. 2001; Zhu & Yu 2009.
Akmonistion[15-17]	Coates & Sequiera 1998; Coates et al. 1998; Coates & Sequeira 2001.
Austroptyctodus[18,19]	Long, 1997; Miles & Young 1977.
Bothriolepis[20-22]	Denison 1978; Young 1986; Young 1988.
Brachyacanthus[23,24]	Watson 1937; Denison 1979.
Brindabellaspis[21, 25-27]	Young 1980, 1986; Burrow & Turner 1998, 1999.
Brochoadmones[28-30]	Bernacsek & Dineley 1977; Gagnier & Wilson 1996; Hanke & Wilson 2006.
Buchanosteus[21,31,32]	Young 1979, 1986; Young et al. 2001.
Campbellodus[18,19]	Miles & Young 1977; Long 1997.
Cassidiceps[29]	Gagnier & Wilson 1996.
Cheiracanthus[23,24,33]	Watson 1937; Miles 1973b; Denison 1979.
Cheirolepis[34-37]	Ørvig 1967; Pearson & Westoll 1979; Arratia & Cloutier 1996; Giles et al. 2015.
Chondrenchelys[38-40]	Moy-Thomas 1935; Lund 1982; Finarelli & Coates 2014.
Cladodoides[41-43]	Gross 1937, 1938; Maisey 2005.
Cladoselache[44-49]	Hussakof & Bryant 1918; Woodward & White 1938; Bendix-Almgreen 1975; Schaeffer 1981; Zangerl 1981; Maisey 2007.
Climatius[23,33,34]	Watson 1937; Ørvig 1967; Miles 1973b.
Cobelodus[49,50]	Zangerl & Case 1976; Maisey 2007.
Coccosteus[8,51]	Stensiö 1963; Miles & Westoll 1968.
Cowralepis[52-54]	Ritchie 2005; Carr et al. 2009; Long et al. 2009.
Culmacanthus[55,56]	Long, 1983; Burrow & Young 2012.
Debeerius[57]	Grogan & Lund 2000.
Diabolepis[58,59]	Chang & Yu 1984; Chang 1995.
Dialipina[60,61]	Schultze 1968; Schultze & Cumbaa 2001.
Dicksonosteus[62,63]	Goujet 1975, 1984.
Diplacanthus[9,23,64]	Watson 1937; Miles 1973a; Gagnier 1996.
Diplocercides[65]	Forey 1998.
Dipterus[66-67]	Parrington 1950; White 1965; Challands 2015.
Doliodus[69-71]	Miller et al. 2003; Turner 2004; Maisey et al. 2009.
Entelognathus[72]	Zhu et al. 2013.
Eurycaraspis[73]	Liu 1991.
Eusthenopteron[74]	Jarvik 1980.

Euthacanthus[23,75]	Watson, 1937; Newman et al. 2011.
Gavinia[76]	Long, 1999.
Gemuendina[77]	Gross 1963.
Gladiobranchus[28,78,79]	Bernacsek & Dineley 1977; Hanke & Davis 2008; Newman et al. 2012.
Glyptolepis[80-83]	Andrews & Westoll 1970; Jarvik 1972; Cloutier & Schultze 1996; Ahlberg 1989.
Gogonasus[84-86]	Long et al. 1997; Long et al. 2006; Holland 2014.
Guiyu[87-89]	Zhu et al. 2009; Qiao & Zhu 2010; Zhu et al. 2012.
Gyracanthides[90,91]	Warren et al. 2000; Turner et al. 2005.
Hamiltonichthys[92]	Maisey, 1989.
Helodus[93]	Moy-Thomas 1936.
Homalacanthus[23,64]	Watson 1937; Gagnier 1996.
Howqualepis[37,94]	Long 1988; Giles et al. 2015.
Incisoscutum[95,96]	Johanson & Smith 2005; Ahlberg et al. 2009.
Ischnacanthus[7,23,33,97]	Watson 1937; Gross 1947; Miles 1973b; Blais et al. 2011.
Jagorina[98]	Stensiö 1969.
Janusiscus[99]	Giles et al. 2015.
Kansasiella[100]	Poplin 1974.
Kathemacanthus[101,102]	Gagnier & Wilson 1996; Hanke & Wilson 2010.
Kentuckia[103,104]	Rayner 1951; Giles & Friedman 2014.
Kujdanowiaspis[98,105]	Stensiö 1969; Dupret 2010.
Latviacanthus[106]	Schultze & Zidek 1982.
Lawrenciella[107]	Hamel & Poplin 2008.
'Ligulalepis'[108,109]	Basden et al. 2000; Basden & Young 2001.
Lunaspis[110,111]	Heintz 1937; Gross 1961.
Lupopsyrus[28]	Bernacsek & Dineley 1977.
Macropetalichthys[20,98, 112-114]	Stensiö 1925, 1969; Gross 1935; Denison 1978; Young, 1978.
Meemannia[115-117]	Zhu et al. 2006; Zhu et al. 2010; Lu et al. 2016.
Mesacanthus[7,23,33]	Watson 1937; Gross 1947; Miles 1973b.
Miguashaia[65,118]	Cloutier 1996; Forey 1998.
Mimipiscis[104,119,120]	Gardiner & Bartram 1977; Gardiner 1984; Giles & Friedman 2014.
Moythomasia[120]	Gardiner 1984.
Obtusacanthus[121]	Hanke & Wilson 2004.
Onychodus[122]	Andrews et al. 2006.
Onychoselache[123,124]	Dick & Maisey 1980; Coates & Gess 2007.
Orthacanthus[47,125]	Schaeffer 1981; Maisey 1983.
Parayunnanolepis[126,127]	Zhang et al. 2001; Zhu et al. 2012.
Parexus[23,128]	Watson 1937; Miles 1973c.
Poracanthodes[24,129]	Valiukevicius 1992; Denison 1979.
Porolepis[80,130]	Jarvik 1972; Clément 2004.
Powichthys[131,132]	Jessen 1975, 1980.

Promesacanthus[133]	Hanke 2008.
Psarolepis[14,127,134-138]	Zhu & Schultze 1997; Yu 1998; Zhu et al. 1999; Zhu & Yu 2009; Zhu et al. 2012; Qu et al. 2013; Qu et al. 2015.
Pterichthyodes[139]	Hemmings 1978.
Ptomacanthus[9,24,140,141]	Miles 1973a; Denison 1979; Brazeau 2009, 2012.
Pucapampella[142-145]	Maisey 2001; Maisey & Anderson 2001; Janvier & Maisey 2010; Maisey & Lane 2010.
Qingmenodus[146,147]	Lu & Zhu 2009; Lu et al. 2016.
Ramirosuarezia[148]	Pradel et al. 2009.
Raynerius[149]	Giles et al. 2015.
Rhamphodopsis[18,19,150]	Miles 1967; Long 1997; Miles & Young 1977.
Romundina[151,152]	Goujet & Young 2004; Giles et al. 2013.
Sparalepis[153]	Choo et al. 2017.
Styloichthys[154-157]	Zhu & Yu 2002, 2004; Friedman 2007; Lu & Zhu 2008.
Tamiobatis[47,158]	Schaeffer 1981; Williams 1998.
Tetanopsyrus[29,159,160]	Gagnier & Wilson 1995; Gagnier et al. 1999; Hanke et al. 2001.
Tristychius[124,161]	Dick 1978; Coates & Gess 2007.
Uranolophus[162,163]	Denison 1968; Campbell & Barwick 1988.
Vernicomacanthus[33]	Miles 1973b.
Youngolepis[164-167]	Chang & Yu 1981; Chang 1982, 1991, 2004.
Yunnanolepis[152,168,169]	Zhu 1996; Zhang 1980; Giles et al. 2013.

Supplementary Note 1: List of Characters

Characters 1–269 taken from ref.117 (in turn based largely on ref.99). The additional characters (c.270–278) are from refs. 149, 170 and 171. Characters 273–275 are new characters. Ordered multistate characters are indicated with an asterisk (*).

Histology

1. **Tessellate prismatic calcified cartilage:**
   0. absent
   1. present

2. **Prismatic calcified cartilage:**
   0. single layered
   1. multi-layered

3. **Perichondral bone:**
   0. present
   1. absent

4. **Extensive endochondral ossification:**

0. absent
1. present

5. Enamel(oid) present on dermal bones and scales:

0. absent
1. present

6. Enamel:

0. single-layered
1. multi-layered

7. Enamel layers:

0. applied directly to one another (ganoine)
1. separated by layers of dentine

8. Pore canal network (sensu Zhu et al. 2010):

The previous formulation of this character referenced an extensive pore canal network. However, this leaves assessment of presence and absence ambiguous based on subjective interpretations of when such a network might be considered ‘extensive’. Instead, we have reformulated this character to capture those instances in which cavities connect with the outer surface of the bone via pores. Changed from ‘?’ to ‘0’ for *Cassidiceps, Euthacanthus*, *Helodus*, *Homalacanthus*, *Ischnacanthus*, *Janusiscus*, *Kathemacanthus*, *Kentuckia*, *Latviacanthus*, ‘*Ligualepis*’, *Mesacanthus*, *Onychodus*, *Pterichthyodes*, *Tristychius*; from ‘1’ to ‘0’ in *Dialipina*, *Poracanthodes*; from ‘0’ to ‘?’ in *Guiyu*, *Jagorina*, *Moythomasia*.

0. absent
1. present

9. Dentinous tissue:

0. absent
1. present

10. Dentine kind:

0. mesodentine
1. semidentine
2. orthodentine

11. Bone cell lacunae in body scale bases:

0. present
1. absent

12. Main dentinous tissue forming fin spine:

Changed from ‘-‘ to ‘0’ in *Pterichthyodes*.

0. osteodentine

1. orthodentine

Squamation

13. Longitudinal scale alignment in fin webs:

Changed from '-' to '0' in *Pterichthyodes*.

0. present
1. absent

14. Differentiated lepidotrichia:

0. absent
1. present

15. Body scale growth pattern:

0. comprising single odontode unit/generation ("monodontode")
1. comprising a complex of multipe odontode generations/units ("polyodontode")

16. Body scale growth concentric:

0. absent
1. present

17. Generations of odontodes:

0. buried
1. areally growing
2. resorbed

18. Body scales with peg-and-socket articulation:

Changed from '?' to '1' in *Styloichthys*.

0. absent
1. present

19. Scale peg:

Changed from '?' to '0' in *Styloichthys*.

0. broad
1. narrow

20. Anterodorsal process on scale:

Changed from '?' to '1' in *Styloichthys*.

0. absent
1. present

21. Body scale profile:

Changed from '?' to '1' in *Styloichthys*.

0. distinct crown and base demarcated by a constriction ("neck")

1. flattened

22. Profile of scales with constriction between crown and base:

0. neck similar in width to crown
1. neck greatly constricted, resulting in anvil-like shape

23. Body scales with bulging base:

Changed from '?' to '0' in *Styloichthys.*

0. absent
1. present

24. Body scales with flattened base:

Changed from '?' to '1' in *Styloichthys*.

0. present
1. absent

25. Basal pore in scales:

0. absent
1. present

26. Flank scale alignment:

Changed from '-' to '?' for *Chondrenchelys*.

0. vertical rows oblique rows or hexagonal
1. rhombic packing
2. disorganised

27. Scute-like ridge scales (basal fulcra):

0. absent
1. present

28. Sensory line canal:

0. perforates scales
1. passes between scales
2. C-shaped scales

Dermal bones of the skull

29. Dermal ornamentation:

Changed from '1' to '3' for *Glyptolepis*, from '3' to '-' for *Onychoselache*, from '3' to '0' for *Psarolepis*.

0. smooth
1. parallel, vermiform ridges
2. concentric ridges
3. tuberculate

30. **Sensory line network:**
0. preserved as open grooves (sulci) in dermal bones
1. sensory lines pass through canals in dermal bones (open as pores)

31. **Sensory canals/grooves:**
0. contained within the thickness of dermal bones
1. contained in prominent ridges on visceral surface of bone

32. **Jugal portion of infraorbital canal joins supramaxillary canal:**
0. present
1. absent

33. **Dermal skull roof:**
0. includes large dermal plates
1. consists of undifferentiated plates or tesserae

34. **Anterior pit line of dermal skull roof:**
Changed from ‘1’ to ‘0’ for *Cheirolepis*.
0. absent
1. present

35. **Tessera morphology:**
0. large interlocking polygonal plates
1. microsquamose, not larger than body squamation

36. **Cranial spines:**
0. absent
1. present, multicuspid
2. present, monocuspid

37. **Extent of dermatocranial cover:**
0. complete
1. incomplete (limited to skull roof)

38. **Openings for endolymphatic ducts in dermal skull roof:**
Changed from ‘0’ to ‘-‘ for *Ischnacanthus*.
0. present
1. absent

39. **Endolymphatic ducts with oblique course through dermal skull bones:**
0. absent
1. present

40. **Endolymphatic duct relationship to median skull roof bone (i.e. nuchal plate):**
0. within median bone
1. on bones flanking the median bone (e.g. paranuchals)

41. **Pineal opening perforation in dermal skull roof:**
0. present
1. absent

42. **Dermal plate associated with pineal eminence or foramen:**
0. contributes to orbital margin
1. plate bordered laterally by skull roofing bones

43. **Series of paired median skull roofing bones that meet at the dorsal midline of the skull (rectilinear skull roof pattern):**
0. absent
1. present

44. **Broad supraorbital vaults:**
0. absent
1. present

45. **Median commissure between supraorbital sensory lines:**
0. absent
1. present

46. **Dermal cranial joint at level of sphenoid-otic junction:**
Changed from '?' to '-' for *Chondrenchelys*.
0. absent
1. present

47. **Otic canal extends through postparietals:**
0. absent
1. present

48. **Number of bones of skull roof lateral to postparietals:**
We have modified this character to take into account the condition in *Powichthys* and lungfishes (new additions to the matrix), which show more than two bones lateral to the postparietals.
0. two
1. one
2. more than two

49. **Suture between paired skull roofing bones (centrals of placoderms; postparietals of osteichthyans):**

0. straight
1. sinusoidal

50. Medial processes of paranuchal wrapping posterolateral corners of nuchal plate:

0. absent
1. present
2. paranuchals precluded from nuchal by centrals
3. no median posterior skull roof bone

51. Paired pits on ventral surface of nuchal plate:

0. absent
1. present

52. Sclerotic ring:

0. absent
1. present

53. Consolidated cheek plates:

0. absent
1. present

54. Cheek plate:

0. undivided
1. divided (i.e., squamosal and preopercular)

55. Subsquamosals in taxa with divided cheek:

Changed from '?' to '-' for *Chondrenchelys*.

0. absent
1. present

56. Preopercular shape:

Changed from '?' to '-' for *Chondrenchelys*.

0. rhombic
1. bar-shaped

57. Vertical canal associated with preopercular/suborbital canal:

0. absent
1. present

58. Enlarged postorbital tessera separate from orbital series:

0. absent
1. present

59. Extent of maxilla along cheek:

0. to posterior margin of cheek
1. cheek bones exclude maxilla from posterior margin of cheek

60. Dermal neck joint:

0. overlap
1. ginglymoid

61. Sensory line scales/plates on head:

0. unspecialized
1. apposed growth
2. paralleling canal
3. semicylindrical C-shaped ring scales

62. Bony hyoidean gill-cover series (branchiostegals):

0. absent
1. present

63. Branchiostegal plate series along ventral margin of lower jaw:

0. absent
1. present

64. Branchiostegal ossifications*:

0. plate-like
1. narrow and ribbon-like
2. filamentous

65. Branchiostegal ossifications:

Changed from ‘1’ to ‘0’ for *Porolepis*, *Gogonasus*.

0. ornamented
1. unornamented

66. Imbricated branchiostegal ossifications:

0. absent
1. present

67. Median gular:

0. absent
1. present

68. Lateral gular:

0. absent
1. present

69. Opercular (submarginal) ossification:

0. absent
1. present

70. Shape of opercular (submarginal) ossification:

0. broad plate that tapers towards its proximal end
1. narrow, rod-shaped

71. Size of lateral gular plates:

0. extending most of length of the lower jaw
1. restricted to the anterior third of the jaw (no longer than the width of three or four branchiostegals)

Ventral hyoid arch and gill skeleton

72. Gill arches:

Changed from '?' to '0' in *Chondrenchelys*.

0. largely restricted to region under braincase
1. extend far posterior to braincase

73. Basihyal:

0. absent
1. present

74. Interhyal:

0. absent
1. present

75. Hypohyal:

0. absent
1. present

76. Endoskeletal urohyal:

0. absent
1. present

Dentition and jaw bones

77. Oral dermal tubercles borne on jaw cartilages or at margins of the mouth:

0. absent
1. present

78. Oral dermal tubercles patterned in organised rows (teeth):

0. absent
1. present

79. Enamel(oid) on teeth:

0. absent
1. present

80. Cap of enameloid restricted to upper part of teeth (acrodin):

0. absent
1. present

81. Tooth whorls:

0. absent
1. present

82. Bases of tooth whorls:

0. single, continuous plate
1. some or all whorls consist of separate tooth units

83. Distribution of tooth whorls:

Changed from ‘1’ to ‘0’ for *Debeerius*; from ‘-‘ to ‘0’ for *Chondrenchelys*.

0. entire length of tooth row
1. restricted to symphysial region

84. Distribution of tooth whorls:

Changed from ‘?’ to ‘0’ for *Helodus*.

0. upper and lower jaws
1. lower jaws only
2. upper jaws only

85. Teeth ankylosed to dermal bones:

0. absent
1. present

86. Plicidentine:

0. absent
1. present

87. Dermal jaw plates on biting surface of jaw cartilages:

0. absent
1. present

88. Maxillary and dentary marginal bones of mouth:

0. absent
1. present

89. **Premaxilla:**

0. extends under orbit
1. restricted anterior to orbit

90. **Maxilla shape:**

0. splint-shaped
1. cleaver-shaped

91. **Pair of tooth plates (anterior supragnathals or vomers) on ethmoidal plate:**

Changed from '1' to '0' for *Onychodus*, *Chondrenchelys*.

0. absent
1. present

92. **Strong posterior flexion of dentary symphysis:**

Changed from '?' to '-' for *Chondrenchelys*.

0. absent
1. present

93. **Extent of infradentaries:**

0. along much of ventral margin of dentary
1. restricted to posterior half of dentary

94. **Coronoid fangs:**

0. absent
1. present

95. **Position of upper mandibular arch cartilage (and associated cheek plate where present):**

0. entirely suborbital
1. with a postorbital extension

96. **Position of mandibular arch articulations:**

Changed from '0' to '1' for *Cheirolepis*; '0' to '?' in *Chondrenchelys*, *Debeerius.*

0. terminal
1. subterminal

97. **Autopalatine and quadrate:**

Changed from '-' to '0' for *Debeerius*.

0. comineralized
1. separate mineralizations

98. **Large otic process of the palatoquadrate:**

Changed from '1' to '?' for *Chondrenchelys*.

0. absent

1. present

99. Insertion area for jaw adductor muscles on palatoquadrate:

0. ventral or medial
1. lateral

100. Palatoquadrate relationship to dermal cheek bones:

0. articulation narrow and restricted
1. broad articulation

101. Palatoquadrate fused with neurocranium:

0. absent
1. present

102. Oblique ridge or groove along medial face of palatoquadrate:

0. absent
1. present

103. Fenestration of palatoquadrate at basipterygoid articulation:

Changed from ‘0’ to ‘-‘ for *Chondrenchelys*.

0. absent
1. present

104. Perforate or fenestrate anterodorsal (metapterygoid) portion of palatoquadrate:

0. absent
1. present

105. Pronounced dorsal process on Meckelian bone or cartilage:

0. absent
1. present

106. Number of coronoids:

0. four or more
1. three or fewer

107. Preglenoid process:

Changed from ‘?’ to ‘1’ for *Helodus*.

0. absent
1. present

108. Jaw articulation located on rearmost extremity of mandible:

0. absent
1. present

Neurocranium and associated dermal ossifications

109. Precerebral fontanelle:
0. absent
1. present

110. Median dermal bone of palate (parasphenoid):
0. absent
1. present

111. Parasphenoid:
0. lozenge-shaped
1. splint-shaped
2. diamond-shaped

112. Multifid anterior margin of parasphenoid denticle plate:
0. absent
1. present

113. Enlarged ascending processes of parasphenoid:
0. absent
1. present

114. Buccohypophysial canal in parasphenoid:
0. single
1. paired

115. Nasal opening(s):
0. dorsal, placed between orbits
1. ventral and anterior to orbit

116. Posterior nostril:
0. separated from orbital fenestra
1. confluent with orbital fenestra

117. Olfactory tracts:
0. short, with olfactory capsules situated close to telencephalon cavity
1. elongate and tubular (much longer than wide)

118. Prominent pre-orbital rostral expansion of the neurocranium:
0. present
1. absent

119. Pronounced sub-ethmoidal keel:

0. absent
1. present

120. Internasal vacuities:

0. absent
1. present

121. Discrete division of the ethmoid and more posterior braincase at the level of the optic tract canal:

0. absent
1. present

122. Position of myodome for superior oblique eye muscles:

0. posterior and dorsal to foramen for nerve II
1. anterior and dorsal to foramen

123. Endoskeletal intracranial joint:

0. absent
1. present

124. Spiracular groove on basicranial surface:

0. absent
1. present

125. Transverse otic process (sometimes referred to as the lateral commissure):

0. present
1. absent

126. Jugular canal*:

In the previous formulation, a ‘short’ jugular canal was described as being anterior to the skeletal labyrinth. However, most taxa with a short jugular canal have this somewhere along the length of the labyrinth rather than exclusively anterior to it. We have therefore modified the terminology to accommodate this pattern. Changed from ‘?’ to ‘2’ for *Chondrenchelys*.

0. long (invested in otic region along length of skeletal labyrinth)
1. short (restricted to short portion of region of skeletal labyrinth, or anterior to it)
2. absent (jugular vein uninvested in otic region)

127. Spiracular groove on lateral commissure:

0. absent
1. present

128. Subpituitary fenestra:

Onychodus changed from ‘?’ to ‘0’.

0. absent
1. present

129. Supraorbital shelf broad with convex lateral margin:

0. absent
1. present

130. Orbit dorsal or facing dorsolaterally, surrounded laterally by endocranium:

0. present
1. absent

131. Eyestalk attachment area:

0. absent
1. present

132. Postorbital process:

Changed from '?' to '1' for *Chondrenchelys*.

0. absent
1. present

133. Canal for jugular in postorbital process:

Changed from '?' to '1' for *Chondrenchelys*.

0. absent
1. present

134. Series of perforations for innervation of supraorbital sensory canal in supraorbital shelf:

0. absent
1. present

135. Extended prehypophysial portion of sphenoid:

0. absent
1. present

136. Narrow interorbital septum, with outer walls in contact along midline forming a single sheet:

The previous formulation of this character distinguished 'narrow' and 'broad' interorbital septa. This lacks precision, and is subject to differing opinions. We therefore have reformulated this character to refer to distinguish cases where the lateral walls of the braincase are separate in the orbital region from those where they join as a single sheet along the midline.

0. absent
1. present

137. **The main trunk of facial nerve (N. VII):**

0. elongate and passes anterolaterally through orbital floor
1. stout, divides within otic capsule at the level of the transverse otic process

138. **Course of hyoid ramus of facial nerve (N. VII) relative to jugular canal:**

0. traverses jugular canal, with separate exit in otic region
1. intersects jugular canal, with exit through posterior jugular foramen

139. **Glossopharyngeal nerve (N. IX) exit:**

0. foramen situated posteroventral to otic capsule and anterior to metotic fissure
1. through metotic fissure

140. **Relationship of cranial endocavity to basisphenoid:**

0. endocavity occupies full depth of sphenoid
1. enodcavity dorsally restricted

141. **Subcranial ridges:**

0. absent
1. present

142. **Ascending basisphenoid pillar pierced by common internal carotid:**

0. absent
1. present

143. **Canal for lateral dorsal aorta within basicranial cartilage:**

0. absent
1. present

144. **Entrance of internal carotids:**

0. through separate openings flanking the hypophyseal opening or recess
1. through a common opening at the central midline of the basicranium

145. **Canal for efferent pseudobranchial artery within basicranial cartilage:**

0. absent
1. present

146. **Position of basal/basipterygoid articulation:**

Changed from ‘1’ to ‘?’ for *Debeerius*.

0. same anteroposterior level as hypophysial opening
1. anterior to hypophysial opening
2. posterior to hypophysial opening

147. **Articulation between neurocanium and palatoquadrate posterodorsal to orbit (suprapterygoid articulation):**

0. absent
1. present

148. Labyrinth cavity:

0. separated from the main neurocranial cavity by a cartilaginous or ossified capsular wall
1. skeletal capsular wall absent

149. Basipterygoid process (basal articulation) with vertically oriented component:

0. absent
1. present

150. Pituitary vein canal:

0. dorsal to level of basipterygoid process
1. flanked posteriorly by basipterygoid process

151. Lateral (**external or horizontal) semicircular canal:**

0. absent
1. present

152. Sinus superior:

0. absent or indistinguishable from union of anterior and posterior canals with saccular chamber
1. present

153. Lateral (external or horizontal) semicircular canal:

Changed from ‘1’ to ‘?’ for *Moythomasia*.

0. joins the vestibular region dorsal to posterior ampulla
1. joins level with posterior ampulla

154. Lateral semicircular canal in dorsal view:

0. medial to path of jugular vein
1. dorsal to jugular vein

155. Lateral cranial canal:

0. absent
1. present

156. Trigemino-facial recess:

Changed from ‘1’ to ‘0’ for *Eusthenopteron*, *Gogonasus*, *Jagorina*, *Ramirosuarezia*; from ‘?’ to ‘0’ for *Psarolepis*, *Styloichthys*; from ‘?’ to ‘1’ for *Tamiobatis*.

0. absent
1. present

157. **Posterior dorsal fontanelle:**

Changed from '?' to '0' for *Chondrenchelys*.

0. absent
1. present

158. **Shape of posterior dorsal fontanelle:**

Changed from '?' to '-' for *Chondrenchelys*.

0. approximately as long as broad
1. much longer than wide, slot-shaped

159. **Synotic tectum:**

Changed from '?' to '-' for *Chondrenchelys*.

0. absent
1. present

160. **Dorsal ridge:**

0. absent
1. present

161. **Shape of median dorsal ridge anterior to endolymphatic fossa:**

0. developed as a squared-off ridge or otherwise ungrooved
1. bears a midline groove

162. **Endolymphatic ducts in neurocranium:**

Changed code from '?' to '1' for *Chondrenchelys*.

0. posteriodorsally angled tubes
1. tubes oriented vertically through median endolymphatic fossa

163. **Position of hyomandibula articulation on neurocranium:**

Changed code from '?' to '1' for *Ligulalepis*.

0. below or anterior to orbit, on ventrolateral angle of braincase
1. on otic capsule, posterior to orbit

164. **Position of hyomandibula articulation relative to structure of skeletal labyrinth:**

0. anterior or lateral to skeletal labyrinth
1. at level of posterior semicircular canal

165. **Hyoid arch articulation on braincase:**

0. single
1. double

166. **Branchial ridges*:**

0. present
1. reduced to vagal process

2. absent (articulation made with bare cranial wall)

167. Craniospinal process:

Changed from ‘?’ to ‘0’ for *Chondrenchelys*.

0. absent
1. present

168. Ventral cranial fissure:

Changed from ‘?’ to ‘0’ for *Chondrenchelys*.

0. absent
1. present

169. Basicranial fenestra:

Changed from ‘?’ to ‘-‘ for *Chondrenchelys*.

0. absent
1. present

170. Metotic (otic-occipital) fissure:

0. absent
1. present

171. Vestibular fontanelle:

Changed from ‘1’ to ‘?’ for *Porolepis*; ‘?’ to ‘0’ for *Chondrenchelys*.

0. absent
1. present

172. Occipital arch wedged in between otic capsules:

Changed from ‘?’ to ‘0’ for *Psarolepis*.

0. absent
1. present

173. Spino-occipital nerve foramina:

0. two or more, aligned horizontally
1. one or two, dorsoventrally offset

174. Ventral notch between parachordals:

0. present or entirely unfused
1. absent

175. Parachordal shape:

0. forming a broad, flat surface as wide as the otic capsules
1. mediolaterally constricted relative to the otic capsules

176. Stalk-shaped parachordal/occipital region:

0. absent
1. present

177. Paired occipital facets:

0. absent
1. present

178. Size of aperture to notochordal canal:

0. much smaller than foramen magnum
1. as large, or larger, than foramen magnum

179. Canal for median dorsal aorta within basicranium:

Changed from '?' to '0' for *Chondrenchelys*.

0. absent
1. present

180. Hypotic lamina (and dorsally directed glossopharyngeal canal):

Changed from '?' to '-' for *Chondrenchelys*.

0. absent
1. present

Paired fins and girdles

181. Macromeric dermal shoulder girdle:

0. present
1. absent

182. Dermal shoulder girdle composition:

0. ventral and dorsal (scapular) components
1. ventral components only

183. Shape of dorsal blade of dermal shoulder girdle (either cleithrum or anterolateral plate):

0. spatulate
1. pointed

184. Dermal shoulder girdle forming a complete ring around the trunk:

0. present
1. absent

185. Pectoral fenestra completely encircled by dermal shoulder armour:

0. present
1. absent

186. Median dorsal plate:

0. absent
1. present

187. Posterior dorsolateral (PDL) plate or equivalent:

0. absent
1. present

188. Pronounced internal median keel on dorsal shoulder girdle (i.e., crista of median dorsal plate):

0. absent
1. present

189. Crista internalis of dermal shoulder girdle:

0. absent
1. present

190. Scapular infundibulum:

0. absent
1. present

191. Scapular process of shoulder endoskeleton:

0. absent
1. present

192. Ventral margin of separate scapular ossification:

0. horizontal
1. deeply angled

193. Cross sectional shape of scapular process:

0. flattened or strongly ovate
1. subcircular

194. Flange on trailing edge of scapulocoracoid:

0. absent
1. present

195. Scapular process with posterodorsal angle:

0. absent
1. present

196. Endoskeletal postbranchial lamina on scapular process:

0. present
1. absent

197. **Mineralisation of internal surface of scapular blade:**

0. mineralised all around
1. unmineralised on internal face forming a hemicylindrical cross-section

198. **Coracoid process:**

0. absent
1. present

199. **Procoracoid mineralisation:**

0. absent
1. present

200. **Fin base articulation on scapulocoracoid:**

0. deeper than wide (stenobasal)
1. wider than deep (eurybasal)

201. **Pectoral fin articulation:**

0. monobasal
1. polybasal

202. **Number of basals in polybasal pectoral fins:**

0. three or more
1. two

203. **Branching radials in paired fins:**

0. absent
1. present

204. **Number of mesomeres in metapterygial axis:**

0. five or fewer
1. seven or more

205. **Biserial pectoral fin endoskeleton:**

0. absent
1. present

206. **Perforate propterygium:**

Changed from '-' to '0' for *Chondrenchelys*.

0. absent
1. present

207. **Filamentous extension of pectoral fin from axillary region:**

0. absent

1. present

208. Pelvic fins:

Changed from ‘?’ to ‘0’ for Galeaspida.

0. absent
1. present

209. Pelvic claspers:

0. absent
1. present

210. Dermal pelvic clasper ossifications:

0. absent
1. present

211. Pectoral fins covered in macromeric dermal armour:

0. absent
1. present

212. Pectoral fin base has large, hemispherical dermal component:

Changed from ‘?’ to ‘0’ for *Styloichthys*.

0. absent
1. present

Axial skeleton including median fins and their supports

213. Dorsal fin spines:

0. absent
1. present

214. Anal fin spine:

0. absent
1. present

215. Paired fin spines:

0. absent
1. present

216. Median fin spine insertion:

0. shallow, not greatly deeper than dermal bones/scales
1. deep

217. Intermediate fin spines:

0. absent

1. present

218. Fin spine cross-section:

0. Round or horseshoe shaped
1. Flat-sided, with rectangular profile

219. Intermediate spines when present:

0. one pair
1. multiple pairs

220. Prepectoral fin spines:

0. absent
1. present

221. Fin spines with ridges:

0. absent
1. present

222. Fin spines with nodes:

0. absent
1. present

223. Fin spines with rows of large retrorse denticles:

0. absent
1. present

224. Expanded spine rib on leading edge of spine:

0. absent
1. present

225. Spine ridges:

0. converging at the distal apex of the spine
1. converging on leading edge of spine

226. Synarcual:

0. absent
1. present

227. Series of thoracic supraneurals:

0. absent
1. present

228. Number of dorsal fins, if present:

0. one

1. two

229. Posterior dorsal fin shape:

0. base approximately as broad as tall, not broader than all of other median fins
1. base much longer than the height of the fin, substantially longer than any of the other dorsal fins

230. Basal plate in dorsal fin:

0. absent
1. present

231. Branching radial structure articulating with dorsal fin basal plate:

0. absent
1. present

232. Anal fin:

0. absent
1. present

233. Basal plate in anal fin:

0. absent
1. present

234. Caudal radials:

0. extend beyond level of body wall and deep into hypochordal lobe
1. radials restricted to axial lobe

235. Supraneurals in axial lobe of caudal fin:

0. absent
1. present

236. Epichordal lepidotrichia in caudal fin:

0. absent
1. present

237. Enamel and pore canals:

0. enamel absent from inner surface of pores
1. enamel lines portions of pore canal

238. Canal-bearing bone of skull roof extends far past posterior margin of parietals:

0. no
1. yes

239. Pineal eminence (in taxa lacking pineal foramen):

0. absent
1. present

240. Position of anterior pitline:

0. on postparietal
1. on parietal

241. Opening in dermal skull roof for spiracular bounded by bones carrying otic canal:

0. absent
1. present

242. Median skull roof bone between postparietals (B-bone):

0. absent
1. present

243. Westoll lines:

0. absent
1. present

244. Preoperculosubmandibular:

0. absent
1. present

245. Hyomandibula:

0. imperforate
1. perforate

246. Urohyal shape:

0. absent
1. vertical plate

247. Maxilla (in taxa with marginal jaw bones):

0. present
1. absent

248. Length of dentary:

0. constitutes a majority of jaw length
1. half the length of jaw or less

249. Labial pit:

Cloutier & Ahlberg 1996; Zhu et al. 2001; Zhu & Yu 2002; Friedman 2007.

0. absent
1. present

250. **Prearticular symphysis:**

Zhu et al. 2001; Zhu & Yu 2002; Friedman 2007.

0. absent
1. present

251. **Mandibular sensory canal:**

0. extends through infradentaries
1. extends through infradentaries and dentary

252. **Extensive flange composed of prearticular and Meckelian bone that extends beyond ventral edge of outer dermal series:**

0. absent
1. present

253. **Posterior coronoid:**

0. similar to anterior coronoids
1. forms expanded coronoid process

254. **Retroarticular process:**

0. absent
1. present

255. **Inturned medial process of premaxilla:**

0. absent
1. present

256. **Anteriorly directed adductor fossae between neurocranium and skull roof:**

0. absent
1. present

257. **Vomerine fangs:**

0. absent
1. present

258. **Number of dermopalatines:**

0. multiple
1. one

259. **Entopterygoids:**

0. separated
1. contact along midline

260. **Rostral tubuli:**

0. absent
1. present

261. Position of anterior nostril:

0. facial
1. at oral margin

262. Posterior nostril*:

0. facial
1. at margin of oral cavity
2. palatal

263. Three large pores (in addition to nostrils) associated with each side of ethmoid (rostral organ):

0. absent
1. present

264. Ventral face of nasal capsule in taxa with mineralized ethmoid*:

0. complete
1. fenestra ventrolateralis
2. entire floor unmineralized

265. Size of profundus canal in postnasal wall:

0. small
1. large

266. Paired pineal and parapineal tracts:

0. absent
1. present

267. Posterior of parasphenoid:

0. restricted to ethmosphenoid region
1. extends to otic region

268. Endoskeletal spiracular canal*:

0. open
1. partial enclosure or spiracular bar
2. complete enclosure in canal

269. Barbed lepidotrichial segments:

0. absent
1. present

Additional characters:

270. Relative position of jugular groove/canal and hyomandibular articulation:

0 hyomandibula dorsal
1 hyomandibula ventral
2 hyomandibula straddles

271. Optic lobes:

0 narrower than cerebellum
1 same width or wider than cerebellum

272. Crus commune of anterior and posterior semicircular canals:

0 dorsal to endocranial roof
1 ventral to endocranial roof

273. Cranial cavity and labyrinth:

New character.
0 widely spaced
1 closely spaced

274. Lateral semicircular canal:

New character.
0 obiquely oriented
1 horizontally oriented

275. Supraotic cavity:

New character.
0 absent
1 present

276. Pelvic girdle with substantial dermal component:

0 present
1 absent

277. Pelvic fin spine:

0 absent
1 present

278. Pelvic fin:

0 monobasal
1 polybasal

Supplementary References

1 Donoghue, P. C. J., Forey, P. L., and Aldridge, R. J. (2000). Conodont affinity and chordate phylogeny. Biolo. Rev. *75*, 191–251.

2 Wang, N.-Z., Donoghue, P. C. J., Smith, M. M., and Sansom, I. J. (2005). Histology of the galeaspid dermoskeleton and endoskeleton, and the origin and early evolution of the vertebrate cranial endoskeleton. J. Vertebr. Paleontol. *25*, 745–756.

3 Gai, Z.-K., Donoghue, P. C. J., Zhu, M., Janvier, P., and Stampanoni, M. (2011). Fossil jawless fish from China foreshadows early jawed vertebrate anatomy. Nature *476*, 324–327.

4 Denison, R. H. (1947). The exoskeleton of *Tremataspis*. Am. J. Sci. *245*, 337–365.

5 Janvier, P. (1985). Les Céphalaspides du Spitsberg: anatomie, phylogénie et systématique des Ostéostracés siluro-dévoniens; revisions des Ostéostracés de la Formation de Wood Bay (Dévonien inférieur du Spitsberg). (Paris: Cahiers de Paléontologie, Section Vertebres, Centre National de la Recherche Scientifique).

6 Sansom, I. J., Miller, C. G., Heward, A., Davies, N. S., Booth, G. A., Fortey, R. A., and Paris, F. (2009). Ordovician fish from the Arabian Peninsula. Palaeontology *52*, 337–342.

7 Gross, W. (1947). Die Agnathen und Acanthodier des Obersilurischen Beyrichienkalks. Palaeontogr. Abt. A *96*, 91–158.

8 Miles, R. S., and Westoll, T. S. (1968). The placoderm fish *Coccosteus cuspidatus* Miller ex Agassiz from the Middle Old Red Sandstone of Scotland. Part I. Descriptive morphology. Trans. Roy. Soc. Edinb. Earth Sci. *67*, 373–476.

9 Miles, R. S. (1973a). Relationships of acanthodians. In: Interrelationships of Fishes, P. H. Greenwood, R. S. Miles and C. Patterson, eds. (London: Academic Press), pp. 63–103.

10 Coates, M. I. (1994). Actinopterygian and acanthodian fishes from the Viséan of East Kirkton, West Lothian, Scotland. Trans. Roy. Soc. Edinb. Earth Sci. *84*, 317–327.

11 Valiukevicius, J. (1995). Acanthodian histology: some significant aspects in taxonomical and phylogenetical research. Geobios M. S. *19*, 157–159.

12 Davis, S. P., Finarelli, J. A., and Coates, M. I. (2012). *Acanthodes* and shark-like conditions in the last common ancestor of modern gnathostomes. Nature *486*, 247–250.

13 Zhu, M., Yu, X.-B., and Ahlberg, P. E. (2001). A primitive sarcopterygian fish with an eyestalk. Nature *410*, 81–84.

14 Zhu, M. and X.-B. Yu (2009). Stem sarcopterygians have primitive polybasal fin articulation. *Bio. Lett. 5*: 372–375.

15 Coates, M. I., and Sequeira, S. E. K. (1998). The braincase of a primitive shark. Trans. Roy. Soc. Edinb. Earth Sci. *89*, 63–85.

16 Coates, M. I., Sequeira, S. E. K., Sansom, I. J., and Smith, M. M. (1998). Spines and tissues of ancient sharks. Nature *396*, 729–730.

17 Coates, M. I., and Sequeira, S. E. K. (2001). A new stethacanthid chondrichthyan from the Lower Carboniferous of Bearsden, Scotland. J. Vertebr. Paleontol. *21*, 438–459.

18 Long, J. A. (1997). Ptyctodontid fishes (Vertebrata, Placodermi) from the Late Devonian Gogo Formation, Western Australia, with a revision of the European genus *Ctenurella* Ørvig, 1960. Geodiversitas *19*, 515–555.

19 Miles, R. S., and Young, G. C. (1977). Placoderm interrelationships reconsidered in the

light of new ptyctodontids from Gogo, Western Australia. In Problems in Vertebrate Evolution, S. M. Andrews, R. S. Miles and A. D. Walker, eds. (London: Academic Press), pp. 123–198.
20 Denison, R. H. (1978). Placodermi. In: Handbook of Paleoichthyology, Vol. 2, H.-P. Schultze, ed. (Stuttgart: Gustav Fischer Verlag), p. 128.
21 Young, G. C. (1986). The relationships of placoderm fishes. Zool. J. Linn. Soc. *88*, 1–57.
22 Young, G. C. (1988). Antiarchs (Placoderm fishes) from the Devonian Aztec Silstone, Southern Victoria Land, Antarctica. Palaeontogr. Abt. A *202*, 1–125.
23 Watson, D. M. S. (1937). The acanthodian fishes. Phil. Trans. R. Soc. Lond. B *228*, 49–146.
24 Denison, R. H. (1979). Acanthodii, (Stuttgart: Gustav Fischer Verlag).
25 Young, G. C. (1980). A new Early Devonian placoderm from New South Wales, Australia, with a discussion of placoderm phylogeny. Palaeontogr. Abt. A *167*, 10–76.
26 Burrow, C. J., and Turner, S. (1998). Devonian placoderm scales from Australia. J. Vertebr. Paleontol. *18*, 677–695.
27 Burrow, C. J., and Turner, S. (1999). A review of placoderm scales, and their significance in placoderm phylogeny. J. Vertebr. Paleontol. *19*, 204–219.
28 Bernacsek, G. M., and Dineley, D. L. (1977). New acanthodians from the Delorme Formation (Lower Devonian) of N. W. T., Canada. Palaeontogr. Abt. A *158*, 1–25.
29 Gagnier, P.-Y., and Wilson, M. V. H. (1995). New evidences on jaw bones and jaw articulations in acanthodians. Geobios M. S. *19*, 137–143.
30 Hanke, G. F., and Wilson, M. V. H. (2006). Anatomy of the Early Devonian acanthodian *Brochoadmones milesi* based on nearly complete body fossils, with comments on the evolution and development of paired fins. J. Vertebr. Paleontol. *26*, 526–537.
31 Young, G. C. (1979). New information on the structure and relationships of *Buchanosteus* (Placodermi: Euarthrodira) from the Early Devonian of New South Wales. Zool. J. Linn. Soc. *66*, 309–352.
32 Young, G. C., Lelièvre, H., and Goujet, D. (2001). Primitive jaw structure in an articulated brachythoracid arthrodire (placoderm fish; Early Devonian) from southeastern Australia. J. Vertebr. Paleontol. *21*, 670–678.
33 Miles, R. S. (1973b). Articulated acanthodian fishes from the Old Red Sandstone of England, with a review of the structure and evolution of the acanthodian shoulder-girdle. Bull. Br. Mus. Nat. Hist. *24*, 111–213.
34 Ørvig, T. (1967). Phylogeny of tooth tissues: evolution of some calcified tissues in early vertebrates. In Structural and Chemical Organization of Teeth, A. Miles, ed. (New York: Academic Press), pp. 45–110.
35 Pearson, D. M., and Westoll, T. S. (1979). The Devonian actinopterygian *Cheirolepis* Agassiz. Trans. Roy. Soc. Edinb. Earth Sci. *70*, 337–399.
36 Arratia, G., and Cloutier, R. (1996). Reassessment of the morphology of *Cheirolepis canadensis* (Actinopterygii). In: Devonian Fishes and Plants of Miguasha, Quebec, Canada, H.-P. Schultze and R. Cloutier, eds. (München: Verlag Dr Friedrich Pfeil), pp. 165–197.
37 Giles, S., Coates, M. I., Garwood, R. J., Brazeau, M. D., Atwood, R., Johanson, Z.,

Friedman, M., and Ruta, M. (2015). Endoskeletal structure in *Cheirolepis* (Osteichthyes, Actinopterygii), an early ray-finned fish. Palaeontology *58*, 849–870.
38 Moy-Thomas, J. A. (1935). The structure and sffinities of *Chondrenchelys problematica* Traquair. Proc. Zool. Soc. Lond. *105*, 391–404 (1935).
39 Lund, R. (1982). *Harpagofututor volsellorhinus* new genus and species (Chondrichthyes, Chondrenchelyiformes) from the Namurian Bear Gulch Limestone, Chondrenchelys problematica and their sexual dimorphism. J. Vertebr. Paleontol. *56*, 938–958.
40 Finarelli, J. A., and Coates, M. I. (2014). *Chondrenchelys problematica* (Traquair, 1888) redescribed: a Lower Carboniferous, eel-like holocephalan from Scotland. Trans. Roy. Soc. Edinb. Earth Sci. *105*, 35–59.
41 Gross, W. (1937). Das Kopfskelett von *Cladodus wildungensis*, 1. Endocranium und Palatoquadratum. Senckenbergiana *19*, 80–107.
42 Gross, W. (1938). Das Kopfskelett von *Cladodus wildungensis* Jaekel. 2, Teil: Der Kieferbogen. Anhang: *Protacrodus vetustus* Jaekel. Senckenbergiana *20*, 123–145.
43 Maisey, J. G. (2005). Braincase of the Upper Devonian shark *Cladodoides wildungensis* (Chondrichthyes, Elasmobranchii), with observations on the braincase in early chondrichthyans. Bull. Am. Mus. Nat. Hist. *288*, 1–103.
44 Hussakof, L., and Bryant, W. L. (1918). Catalog of the fossil fishes in the museum of the Buffalo Society of Natural Sciences. Bull. Buffalo Soc. Nat. Sci. *12*, 1–198.
45 Woodward, A. S., and White, E. I. (1938). The dermal tubercles of the Upper Devonian shark, *Cladoselache*. Ann. Mag. Nat. Hist. *11*, 367–368.
46 Bendix-Almgreen, S. E. (1975). The paired fins and shoulder girdle in *Cladoselache*, their morphology and phyletic significance. In: Problèmes actuels de Paléontologie-Evolution des Vertébrés, Volume 218, J. P. Lehman, ed. (Paris: Colloques Internationaux du Centre National de la Recherche Scientifique), pp. 111–123.
47 Schaeffer, B. (1981). The xenacanth shark neurocranium, with comments on elasmobranch monophyly. Bull. Am. Mus. Nat. Hist. *169*, 1–66.
48 Zangerl, R. (1981). Handbook of Paleoichthyology. Volume 3A: Chondrichthyes I (Paleozoic Elasmobranchii), (Stuttgart: Gustav Fischer Verlag).
49 Maisey, J. G. (2007). The braincase in Paleozoic symmoriiform and cladoselachian sharks. Bull. Am. Mus. Nat. Hist. *307*, 1–122.
50 Zangerl, R., and Case, G. R. (1976). *Cobelodus aculeatus* (Cope), an snacanthous shark from Pennsylvanian black shales of North America. Palaeontogr. Abt. A *154*, 107–157.
51 Stensiö, E.A. (1963). Anatomical studies on the arthrodiran head. Part 1. Preface, geological and geographical distribution, the organization of the head in the Dolichothoraci, Coccosteomorphi and Pachyosteomorphi. Taxonomic appendix. Kungl. Svenska vetenskapsakademiens handlingar *9*, 1–419.
52 Ritchie, A. (2005). *Cowralepis*, a new genus of phyllolepid fish (Pisces, Placodermi) from the Late Middle Devonian of New South Wales, Australia. Proc. Linn. Soc. N. S. W. *126*, 215–259.
53 Carr, R. K., Johanson, Z., and Ritchie, A. (2009). The phyllolepid placoderm *Cowralepis mclachlani*: insights into the evolution of feeding mechanisms in jawed vertebrates. J. Morphol. *270*, 775–804.
54 Long, J. A., Trinajstic, K., and Johanson, Z. (2009). Devonian arthrodire embryos and the

origin of internal fertilization in vertebrates. Nature *457*, 1124–1127.
55 Long, J. A. (1983). A new diplacanthoid acanthodian from the Late Devonian of Victoria. Mem. Assoc. Australas. Palaeontol. *1*, 51–65.
56 Burrow, C. J., and Young, G. C. (2012). New information on *Culmacanthus* (Acanthodii: Diplacanthiformes) from the ?Early–Middle Devonian of Southeastern Australia. Proc. Linn. Soc. N. S. W. *134*, 21–29.
57 Grogan, E. D., and Lund, R. (2000). *Debeerius ellefseni* (Fam. nov., gen. nov., spec. nov.), an autodiastylic chondrichthyan from the Mississippian bear gulch limestone of Montana (USA), the relationships of the Chondrichthyes, and comments on gnathostome evolution. J. Morphol. *243*, 219–245.
58 Chang, M.-M., and Yu, X.-B. (1984). Structure and phylogenetic significance of *Diabolichthys speratus* gen. et sp. nov., a new dipnoan-like form from the Lower Devonian of eastern Yunnan, China. Proc. Linn. Soc. N. S. W. *107*, 171–184.
59 Chang, M.-M. (1995). *Diabolepis* and its bearing on the relationships between porolepiforms and dipnoans. Bull. Mus. Natn. Hist. Nat., Paris 4e sér., Section C *1-4*, 235–268.
60 Schultze, H.-P. (1968). Palaeoniscoidea-schuppen aus dem Unterdevon Australiens und Kansas und aus dem Mitteldevon Spitzbergens. Bull. Br. Mus. Nat. Hist. *16*, 343–368.
61 Schultze, H.-P., and Cumbaa, S. L. (2001). *Dialipina* and the characters of basal actinopterygians. In: Major Events in Early Vertebrate Evolution: Palaeontology, Phylogeny, Genetics and Development, P. E. Ahlberg, ed. (London: Taylor & Francis), pp. 315–332.
62 Goujet, D. (1975). *Dicksonosteus*, un nouvel arthrodire du Dévonien du Spitsberg remarques sur le squelette visceral des Dolichothoraci. In Problèmes actuels de Paléontologie-Evolution des Vertébrés, Volume 218, J. P. Lehman, ed. (Paris: Colloques Internationaux du Centre National de la Recherche Scientifique), pp. 81–99.
63 Goujet, D. (1984). Les poissons placodermes du Spitsberg. Arthrodires Dolichothoraci de la Formation de Wood Bay (Dévonien inférieur), Volume 15, (Paris: Cahiers de Paléontologie, Section Vertebrés, Centre national de la Recherche scientifique).
64 Gagnier, P.-Y. (1996). Acanthodii. In Devonian Fishes and Plants of Miguasha, Quebec, Canada, H.-P. Schultze and R. Cloutier, eds. (München: Verlag Dr. Friedrich Pfeil), pp. 149–164.
65 Forey, P. L. (1998). History of the Coelacanth Fishes, (London: Chapman & Hall).
66 Parrington, F. R. (1950). The skull of *Dipterus*. Ann. Mag. Nat. Hist. *12*, 534–547.
67 White, E. I. (1965). The head of *Dipterus valenciennesi* Sedgewick & Murchison. Bull. Br. Mus. Nat. Hist. *11*, 1–45.
68 Challands, T. J., and Johanson, Z. (2015). The cranial endocast of the Middle Devonian dipnoan *Dipterus valenciennesi* and a fossilized dipnoan otoconial mass. Pap. Palaeontol. *1*, 289–317.
69 Miller, R. F., Cloutier, R., and Turner, S. (2003). The oldest articulated chondrichthyan from the Early Devonian peroid. Nature *425*, 501–504.
70 Turner, S. (2004). Early vertebrates: analysis from microfossil evidence. In: Recent Advances in the Origin and Early Radiation of Vertebrates, G. Arratia, M. V. H. Wilson and R. Cloutier, eds. (München: Verlag Dr. Friedrich Pfeil), pp. 67–94.

71 Maisey, J. G., Miller, R., and Turner, S. (2009). The braincase of the chondrichthyan *Doliodus* from the Lower Devonian Campbellton Formation of New Brunswick, Canada. Acta Zool. *90*, 109–122.

72 Zhu, M., Yu, X.-B., Ahlberg, P. E., Choo, B., Lu, J., Qiao, T., Qu, Q.-M., Zhao, W.-J., Jia, L.-T., Blom, H., et al. (2013). A Silurian placoderm with osteichthyan-like marginal jaw bones. Nature *502*, 188–193.

73 Liu, Y.-H. (1991). On a new petalichthyid, *Eurycaraspis incilis* gen. et sp. nov., from the Middle Devonian of Zhanyi, Yunnan. In: Early Vertebrates and Related Problems of Evolutionary Biology, M.-M. Chang, Y.-H. Liu and G.-R. Zhang, eds. (Beijing: Science Press), pp. 139–177.

74 Jarvik, E. (1980). Basic Structure and Evolution of Vertebrates, Vol. 1, (London: Academic Press).

75 Newman, M. J., Davidson, R. G., den Blaauwen, J. L., and Burrow, C. J. (2011). The Early Devonian Acanthodian *Euthacanthus gracilis* from the Midland Valley of Scotland. Scot. J. Geol. *47*, 101–111.

76 Long, J. A. (1999). A new genus of fossil coelacanth (Osteichthyes: Coelacanthiformes) from the Middle Devonian of southeastern Australia. Rec. West. Aust. Mus. Suppl. *57*, 37–53.

77 Gross, W. (1963). *Gemuendina stuertzi* Traquair. Notizbl. hess. Landesamt. Bodenforsch. *91*, 36–73.

78 Hanke, G. F., and Davis, S. P. (2008). Redescription of the acanthodian *Gladiobranchus probaton* Bernacsek & Dineley, 1977, and comments on diplacanthid relationships. Geodiversitas *30*, 303–330.

79 Newman, M. J., Davidson, R. G., Blaauwen, J. L. D., and Burrow, C. J. (2012). The Early Devonian Acanthodian *Uraniacanthus curtus* (Powrie, 1870) n. comb. from the Midland Valley of Scotland. Geodiversitas *34*, 739–759.

80 Andrews, S. M., and Westoll, T. S. (1970). The postcranial skeleton of rhipidistian fishes excluding *Eusthenopteron*. Trans. Roy. Soc. Edinb. Earth Sci. *68*, 391–486.

81 Jarvik, E. (1972). Middle and Upper Devonian Porolepiformes from East Greenland with special reference to *Glyptolepis groenlandica* n. sp., and a discussion on the structure of the head in the Porolepiformes. Meddel. Grønl. *187*, 1–307.

82 Cloutier, R., and Ahlberg, P. E. (1996). Morphology, characters, and the interrelationships of basal sarcopterygians. In: Interrelationships of Fishes, M. L. J. Stiasnny, L. R. Parenti and G. D. Johnson, eds. (San Diego: Academic Press), pp. 445–479.

83 Ahlberg, P. E. (1989). The anatomy and phylogeny of porolepiform fishes, with special reference to *Glyptolepis*. (University of Cambridge).

84 Long, J. A., Barwick, R. E., and Campbell, K. S. W. (1997). Osteology and functional morphology of the osteolepiform fish *Gogonasus andrewsae* Long, 1985, from the Upper Devonian Gogo Formation, Western Australia. Rec. West. Aust. Mus. Suppl. *53*, 1–89.

85 Long, J. A., Young, G. C., Holland, T., Senden, T. J., and Fitzgerald, E. M. G. (2006). An exceptional Devonian fish from Australia sheds light on tetrapod origins. Nature *444*, 199–202.

86 Holland, T. (2014). The endocranial anatomy of *Gogonasus andrewsae* Long, 1985 revealed through micro CT-scanning. Earth Environ. Sci. Trans. R. Soc. Edinb. *105*, 9–34.

87 Zhu, M., Zhao, W.-J., Jia, L.-T., Lu, J., Qiao, T., and Qu, Q.-M. (2009). The oldest articulated osteichthyan reveals mosaic gnathostome characters. Nature *458*, 469–474.
88 Qiao, T., and Zhu, M. (2010). Cranial morphology of the Silurian sarcopterygian *Guiyu oneiros* (Gnathostomata: Osteichthyes). Sci. China. Earth Sci. *53*, 1836–1848.
89 Zhu, M., X.-B. Yu, Choo, B., Qu, Q.-M., Jia, L.-T., Zhao, W.-J., Qiao, T., and Lu, J. (2012). Fossil fishes from China provide first evidence of dermal pelvic girdles in osteichthyans. PLoS One *7*: e35103.
90 Warren, A., Currie, B. P., Burrow, C., and Turner, S. (2000). A redescription and reinterpretation of *Gyracanthides murrayi* Woodward 1906 (Acanthodii, Gyracanthidae) from the Lower Carboniferous of the Mansfield Basin, Victoria, Australia. J. Vertebr. Paleontol. *20*, 225–242.
91 Turner, S., Burrow, C. J., and Warren, A. (2005). *Gyracanthides hawkinsi* sp. nov. (Acanthodii, Gyracanthidae) from the Lower Carboniferous of Queensland, Australia, with a review of gyracanthid Taxa. Palaeontology *48*, 963–1006.
92 Maisey, J.G. (1989). *Hamiltonichthys mapesi*, g. & sp. nov. (Chondrichthyes; Elasmobranchii), from the Upper Pennsylvanian of Kansas. Am. Mus. Novit. *2931*, 1–42.
93 Moy-Thomas, M.A. (1936). On the structure and affinities of the Carboniferous Cochliodont *Helodus simplex*. Geol. Mag. *73*, 488–503.
94 Long, J. A. (1988). New palaeoniscoid fishes from the Late Devonian and Early Carboniferous of Victoria. Mem. Assoc. Australas. Palaeontol. *7*, 1–64.
95 Johanson, Z., and Smith, M. M. (2005). Origin and evolution of gnathostome dentitions: a question of teeth and pharyngeal denticles in placoderms. Biological Reviews *80*, 303–345.
96 Ahlberg, P., Trinajstic, K., Johanson, Z., and Long, J. A. (2009). Pelvic claspers confirm chondrichthyan-like internal fertilization in arthrodires. Nature *460*, 888–889.
97 Blais, S. A., MacKenzie, L. A., and Wilson, M. V. H. (2011). Tooth-like scales in Early Devonian eugnathostomes and the 'outside-in' hypothesis for the origins of teeth in vertebrates. J. Vertebr. Paleontol. *31*, 1189–1199.
98 Stensiö, E. (1969). Elasmobranchiomorphi Placodermata Arthrodires. In: Traité de Paléontologie, Volume 4, J. Piveteau, ed. (Paris: Masson), pp. 71–692.
99 Giles, S., Friedman, M., and Brazeau, M.D. (2015). Osteichthyan-like cranial conditions in an Early Devonian stem gnathostome. Nature *520*, 82–85.
100 Poplin, C. (1974). Étude de quelques paléoniscidés Pennsylvaniens du Kansas. (Paris: Éditions du Centre national de la recherche scientifique).
101 Gagnier, P.-Y., and Wilson, M. V. H. (1996). An unusual acanthodian from northern Canada: revision of *Brochoadmones milesi*. Mod. Geol. *20*, 235–251.
102 Hanke, G. F., and Wilson, M. V. H. (2010). The putative stem-group chondrichthyans *Kathemacanthus* and *Seretolepis* from the Lower Devonian MOTH locality, Mackenzie Mountains, Canada. In: Morphology, Phylogeny and Paleobiogeography of Fossil Fishes, D. K. Elliott, J. G. Maisey, X.-B. Yu and D.-S. Miao, eds. (München: Verlag Dr. Friedrich Pfeil), pp. 159–182.
103 Rayner, D. H. (1951). On the cranial structure of an early palaeoniscid, *Kentuckia* gen. nov. Trans. Roy. Soc. Edinb. Earth Sci. *62*, 53–83.
104 Giles, S., and Friedman, M. (2014). Virtual reconstruction of endocast anatomy in early

ray-finned fishes (Osteichthyes, Actinopterygii). J. Vertebr. Paleontol. *88*, 636–651.
105 Dupret, V. (2010). Revision of the genus *Kujdanowiaspis* Stensiö, 1942 (Placodermi, Arthrodira, "Actinolepida") from the Lower Devonian of Podolia (Ukraine). Geodiversitas *32*, 5–63.
106 Schultze, H.-P., and Zidek, J. (1982). Ein primitiver Acanthodier (Pisces) aus dem Unterdevon Lettlands. Paläont. Z. *56*, 95–105.
107 Hamel, M.-H., and Poplin, C. (2008). The braincase anatomy of *Lawrenciella schafferi*, actinopterygian from the Upper Carboniferous of Kansas (USA). J. Vertebr. Paleontol. *28*, 989–1006.
108 Basden, A. M., Young, G. C., Coates, M. I., and Ritchie, A. (2000). The most primitive osteichthyan braincase? Nature *403*, 185–188.
109 Basden, A. M., and Young, G. C. (2001). A primitive actinopterygian neurocranium from the Early Devonian of southeastern Australia. J. Vertebr. Paleontol. *21*, 754–766.
110 Heintz, A. (1937). Die Downtonischen und Devonischen Vertebraten von Spitzbergen VI. *Lunaspis*-arten aus dem Devon Spitzbergens. Skr. Svalb. og Ishavet. *72*, 1–23.
111 Gross, W. (1961). *Lunaspis broilii* und *Lunaspis heroldi* aus dem Hunsrückschiefer (Unterdevon, Rheinland). Notizbl. hess. Landesamt. Bodenforsch. *89*, 17–43.
112 Stensio, E. (1925). On the head of the macropetalichthyids with certain remarks on the head of the other arthrodires. Publ. Field. Mus. (Geol.) *4*, 87–197.
113 Gross, W. (1935). Histologische Studien am Aussenskelett fossiler Agnathen und Fische. Palaeontogr. Abt. A *83*, 1–60.
114 Young, G. C. (1978). A new Early Devonian petalichthyid fish from the Taemas/Wee Jasper region of New South Wales. Alcheringa *2*, 103–116.
115 Zhu, M., Yu, X.-B., Wang, W., Zhao, W.-J., and Jia, L.-T. (2006). A primitive fish provides key characters bearing on deep osteichthyan phylogeny. Nature *441*, 77–80.
116 Zhu, M., Wang, W., and Yu, X.-B. (2010). *Meemannia eos*, a basal sarcopterygian fish from the Lower Devonian of China – expanded description and significance. In: Morphology, Phylogeny and Paleobiogeography of Fossil Fishes, D. K. Elliott, J. G. Maisey, X.-B. Yu and D.-S. Miao, eds. (München: Verlag Dr. Friedrich Pfeil), pp. 199–214.
117 Lu, J., Giles, S. Friedman, M. den Blaauwen, J. L. and Zhu, M. (2016). The oldest actinopterygian Highlights the cryptic early history of the hyperdiverse ray-finned fishes. *Curr. Biol. 26*: 1602–1608.
118 Cloutier, R. (1996). The primitive actinistian *Miguashaia bureaui* Schultze (Sarcopterygii). In Devonian Fishes and Plants of Miguasha, Quebec, Canada, H.-P. Schultze and R. Cloutier, eds. (München: Verlag Dr. Freidrich Pfeil,), pp. 227–247.
119 Gardiner, B.G., and Bartram, A. W. H. (1977). The homologies of ventral cranial fissures in osteichthyans. In Problems in Vertebrate Evolution, S.M. Andrews, R. S. Miles and A. D. Walker, eds. (London: Academic Press), pp. 227–245.
120 Gardiner, B. G. (1984). The relationships of the palaeoniscid fishes, a review based on new specimens of *Mimia* and *Moythomasia* from the Upper Devonian of Western Australia. Bull. Br. Mus. Nat. Hist. *37*, 173–428.
121 Hanke, G. F., and Wilson, M. V. H. (2004). New teleostome fishes and acanthodian systematics. In Recent Advances in the Origin and Early Radiation of Vertebrates, G.

Arratia, M. V. H. Wilson and R. Cloutier, eds. (München: Verlag Dr. Friedrich Pfeil), pp. 189–216.

122 Andrews, S. M., Long, J. A., Ahlberg, P. E., Barwick, R., and Campbell, K. S. W. (2006). The structure of the sarcopterygian *Onychodus jandemarrai* n. sp. from Gogo, Western Australia: with a functional interpretation of the skeleton. Trans. Roy. Soc. Edinb. Earth Sci. *96*, 197–307.

123 Dick, J. R. F., and Maisey, J. G. (1980). The Scottish Lower Carboniferous shark *Onychoselache traquairi*. Palaeontology *23*, 363–374.

124 Coates, M. I., and Gess, R. W. (2007). A new reconstruction of *Onychoselache traquairi*, comments on early chondrichthyan pectoral girdles and hybodontiform phylogeny. Palaeontology *50*, 1421–1446.

125 Maisey, J. G. (1983). Cranial anatomy of *Hybodus basanus* Egerton from the Lower Cretaceous of England. Am. Mus. Novit. *2758*, 1–64.

126 Zhang, G.-R., Wang, J.-Q., and Wang, N.-Z. (2001). The structure of pectoral fin and tail of Yunnanolepidoidei, with a discussion of the pectoral fin of chuchinolepids. Vertebrat. PalAsiatic. *39*, 1–13.

127 Zhu, M., Yu, X.-B., Choo, B., Wang, J.-Q., and Jia, L.-T. (2012). An antiarch placoderm shows that pelvic girdles arose at the root of jawed vertebrates. Biol. Lett. *8*, 453–456.

128 Miles, R. S. (1973c). An actinolepid arthrodire from the Lower Devonian Peel Sound Formation, Prince of Wales Island. Palaeontogr. Abt. A *143*, 109–118.

129 Valiukevicius, J. (1992). First articulated *Poracanthodes* from the Lower Devonian of Severnaya Zemlya. In: Fossil Fishes as Living Animals, E. Mark-Kurik, ed. (Tallinn: Academy of Sciences of Estonia), pp. 193–213.

130 Clément, G. (2004). Nouvelles données anatomiques et morphologie générale des "Porolepididae" (Diplnomorpha, Sarcopterygii). Rev. Paléobiol. *9*, 193–211.

131 Jessen, H. L. (1975). A new choanate fish, *Powichthys thorsteinssoni* n.g., n.sp., from the early Lower Devonian of the Canadian Arctic Archipelago. In Problèmes actuels de Paléontologie-Evolution des Vertébrés, Volume 218, J. P. Lehman, ed. (Paris: Colloques Internationaux du Centre National de la Recherche Scientifique), pp. 213–222.

132 Jessen, H. L. (1980). Lower Devonian Porolepiformes from the Canadian Arctic with special reference to *Powichthys thorsteinssoni* Jessen. Palaeontogr. Abt. A *167*, 180–214.

133 Hanke, G. F. (2008). *Promesacanthus eppleri* n. gen., n. sp., a mesacanthid (Acanthodii, Acanthodiformes) from the Lower Devonian of northern Canada. Geodiversitas *30*, 287–302.

134 Zhu, M., and Schultze, H.-P. (1997). The oldest sarcopterygian fish. Lethaia *30*, 293–304.

135 Yu, X.-B. (1998). A new porolepiform-like fish, *Psarolepis romeri*, gen. et sp. nov. (Sarcopterygii, Osteichthyes) from the Lower Devonian of Yunnan, China. J. Vertebr. Paleontol. *18*, 261–274.

136 Zhu, M., Yu, X.-B., and Janvier, P. (1999). A primitive fossil fish sheds light on the origin of bony fishes. Nature *397*, 607–610.

137 Qu, Q.-M., Zhu, M., and Wang, W. (2013). Scales and dermal skeletal histology of an early bony fish *Psarolepis romeri* and their bearing on the evolution of rhombic scales and hard tissues. PLoS One *8*, e61485.

138 Qu, Q.-M., Haitina, T., Zhu, M. and Ahlberg, P. E. (2015). New genomic and fossil data

illuminate the origin of enamel. Nature *526*: 108–111.
139 Hemmings, S. K. (1978). The Old Red Sandstone antiarchs of Scotland: *Pterichthyodes* and *Microbrachius*. Palaeontogr. Soc. (Monogr.) *131*, 1–64.
140 Brazeau, M. D. (2009). The braincase and jaws of a Devonian 'acanthodian' and modern gnathostome origins. Nature *457*, 305–308.
141 Brazeau, M. D. (2012). A revision of the anatomy of the Early Devonian jawed vertebrate *Ptomacanthus anglicus* Miles. Palaeontology *55*, 355–367.
142 Maisey, J. G. (2001). A primitive chondrichthyan braincase from the Middle Devonian of Bolivia. In: Major Events in Early Vertebrate Evolution: Palaeontology, Phylogeny, Genetics and Development, P. E. Ahlberg, ed. (London: Taylor & Francis), pp. 263–288.
143 Maisey, J. G., and Anderson, M. E. (2001). A primitive chondrichthyan braincase from the Early Devonian of South Africa. J. Vertebr. Paleontol. *21*, 702–713.
144 Janvier, P., and Maisey, J. G. (2010). The Devonian vertebrates of South America and their biogeographical relationships. In Morphology, Phylogeny and Paleobiogeography of Fossil Fishes, D. K. Elliott, J. G. Maisey, X.-B. Yu and D.-S. Miao, eds. (München: Verlag Dr. Friedrich Pfeil), pp. 431–459.
145 Maisey, J. G., and Lane, J. A. (2010). Labyrinth morphology and the evolution of low-frequency phonoreception in elasmobranchs. C. R. Palevol. *9*, 289–309.
146 Lu, J. and M. Zhu (2010). An onychodont fish (Osteichthyes, Sarcopterygii) from the Early Devonian of China, and the evolution of the Onychodontiformes. Proc. R. Soc. B. *277*: 293–299.
147 Lu, J., M. Zhu, Ahlberg, P. E., Qiao, T. Zhu, Y. A., Zhao, W.-J., Jia, L.-T. (2016). A Devonian predatory fish provides insights into the early evolution of modern sarcopterygians. Sci. Adv. *2*: e1600154.
148 Pradel, A., Maisey, J. G., Tafforeau, P., and Janvier, P. (2009). An enigmatic gnathostome vertebrate skull from the Middle Devonian of Bolivia. Acta Zool. *90*, 123–133.
149 Giles, S., Darras, L., Clément, G., Blieck, A., and Friedman, M. (2015c). An exceptionally preserved Late Devonian actinopterygian provides a new model for primitive cranial anatomy in ray-finned fishes. Proc. R. Soc. B *282*: 20151485.
150 Miles, R. S. (1967). Observations on the ptyctodont fish, *Rhamphodopsis* Watson. Zool. J. Linn. Soc. *47*, 99–120.
151 Goujet, D., and Young, G. C. (2004). Placoderm anatomy and phylogeny: new insights. In Recent Advances in the Origin and Early Radiation of Vertebrates, G. Arratia, M. V. H. Wilson and R. Cloutier, eds. (München: Verlag Dr. Friedrich Pfeil), pp. 109–126.
152 Giles, S., Rücklin, M., and Donoghue, P. C. J. (2013). Histology of "placoderm" dermal skeletons: implications for the nature of the ancestral gnathostome. J. Morphol. *274*, 627–644.
153 Choo, B., M. Zhu, et al. (2017). A new osteichthyan from the late Silurian of Yunnan, China. PLoS One *12*: e0170929.
154 Zhu, M., and Yu, X.-B. (2002). A primitive fish close to the common ancestor of tetrapods and lungfish. Nature *418*, 767–770.
155 Zhu, M., and Yu, X.-B. (2004). Lower jaw character transitions among major sarcopterygian groups-a survery based on new materials from Yunnan, China. In Recent Advances in the Origin and Early Radiation of Vertebrates, G. Arratia, M. V. H. Wilson

and R. Cloutier, eds. (München: Verlag Dr. Friedrich Pfeil), pp. 271–286.
156 Friedman, M. (2007). *Styloichthys* as the oldest coelacanth: implications for early osteichthyan interrelationships. J. Syst. Palaeontol. *5*, 289–343.
157 Lu, J., and Zhu, M. (2008). An Early Devonian (Pragian) sarcopterygian from Zhaotong, Yunnan, China. Vertebr. PalAsiat. *46*, 161–170.
158 Williams, M. E. (1998). A new specimen of *Tamiobatis vetustus* (Chondrichthyes, Ctenacanthoidea) from the Late Devonian Cleveland Shale of Ohio. J. Vertebr. Paleontol. *18*, 251–260.
159 Gagnier, P.-Y., Hanke, G. F., and Wilson, M. V. H. (1999). *Tetanopsyrus lindoei* gen. et sp. nov., an Early Devonian acanthodian from the Northwest Territories, Canada. Acta Geol. Pol. *49*, 81–96.
160 Hanke, G. F., Davis, S. P., and Wilson, M. V. H. (2001). New species of the acanthodian genus *Tetanopsyrus* from northern Canada, and comments on related taxa. J. Vertebr. Paleontol. *21*, 740–753.
161 Dick, J. R. F. (1978). On the Carboniferous shark *Tristychius arcuatus* Agassiz from Scotland. Trans. Roy. Soc. Edinb. Earth Sci. *70*, 63–109.
162 Denison, R. H. (1968). Early Devonian lungfishes from Wyoming, Utah, and Idaho. Fieldiana Geol. *17)*, 353–413.
163 Campbell, K.S. W., and Barwick, R. E. (1988). *Uranolophus*: a reappraisal of a primitive dipnoan. Mem. Assoc. Australas. Palaeontol. *7*, 87–144.
164 Chang, M.-M., and Yu, X.-B. (1981). A new crossopterygian, *Youngolepis praecursor*, gen. et sp. nov., from Lower Devonian of E. Yunnan, China. Sci. Sin. *24*, 89–97.
165 Chang, M.-M. (1982). The braincase of *Youngolepis*, a Lower Devonian crossopterygian from Yunnan, south-western China, (Stockholm: University of Stockholm, Department of Geology).
166 Chang, M.-M. (1991). Head exoskeleton and shoulder girdle of *Youngolepis*. In Early Vertebrates and Related Problems of Evolutionary Biology, M.-M. Chang, Y.-H. Liu and G.-R. Zhang, eds. (Beijing: Science Press), pp. 355–378.
167 Chang, M.-M. (2004). Synapomorphies and scenarios - more characters of *Youngolepis* betraying its affinity to the Dipnoi. In: Recent Advances in the Origin and Early Radiation of Vertebrates, G. Arratia, M. V. H. Wilson and R. Cloutier, eds. (München: Verlag Dr. Friedrich Pfeil), pp. 665–686.
168 Zhu, M. (1996). The phylogeny of the Antiarcha (Placodermi, Pisces), with the description of Early Devonian antiarchs from Qujing, Yunnan, China. Bull. Mus. Natl. Hist. Nat. *18*, 233–347.
169 Zhang, M.-M. (1980). Preliminary note on a Lower Devonian antiarch from Yunnan, China. Vertebrat. PalAsiatic. *18*, 179–190.
170 Brazeau, M. D. and de Winter, V. (2015). The hyoid arch and braincase anatomy of Acanthodes support chondrichthyan affinity of ‘acanthodians’. Proc. R. Soc. B *282*: 20152210.
171 Long, J. A., Mark-Kurik, E., Johanson, Z., Lee, M. S. Y., Young, G. C., Zhu, M., Ahlberg,P. E., Newman, M., Jones, R., den Blaauwen, J., Choo, B., and Trinajsticet K., (2015). Copulation in antiarch placoderms and the origin of gnathostome internal fertilization. Nature *517*: 196–199.
