# Supplementary material for: A new stem sarcopterygian illuminates patterns of character evolution in early bony fishes
Source: Nat Commun. 2017 Dec 5;8:1932. doi: 10.1038/s41467-017-01801-z (PMC5715141; doi:10.1038/s41467-017-01801-z)
Supplement: Supplementary file 3 — Description of Additional Supplementary Files [file 41467_2017_1801_MOESM3_ESM.pdf]

File Name: Supplementary Data 1
Description: A large-format PDF of a phylogenetic tree depicting all character state transformations on internal nodes

File Name: Supplementary Data 2
Description: A Nexus format file containing all phylogenetic character data
