# Supplementary material for: A new stem sarcopterygian illuminates patterns of character evolution in early bony fishes
Source: Nat Commun. 2017 Dec 5;8:1932. doi: 10.1038/s41467-017-01801-z (PMC5715141; doi:10.1038/s41467-017-01801-z)

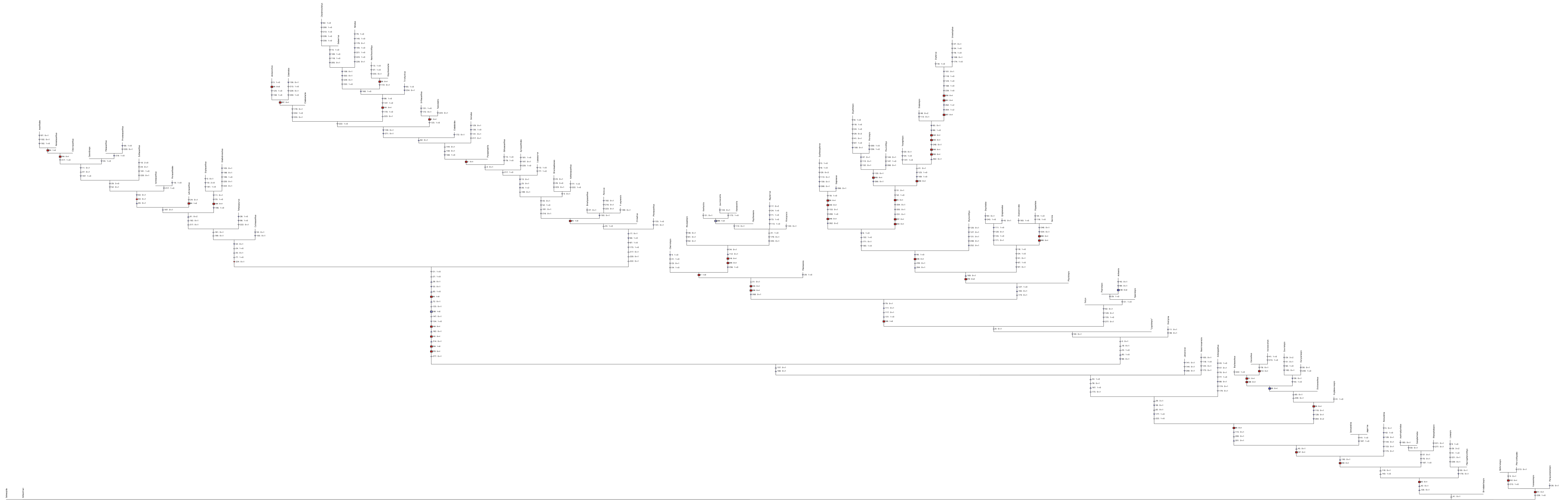

Supplement: Supplementary file 4 — Supplementary Data 1 [file 41467_2017_1801_MOESM4_ESM.pdf]
